# Breast cancer recurrence prediction with ensemble methods and cost-sensitive learning

**DOI:** 10.1515/med-2021-0282

**Published:** 2021-05-13

**Authors:** Pei-Tse Yang, Wen-Shuo Wu, Chia-Chun Wu, Yi-Nuo Shih, Chung-Ho Hsieh, Jia-Lien Hsu

**Affiliations:** Department of Computer Science and Information Engineering, Fu Jen Catholic University, New Taipei City, Taiwan, Republic of China; Department of Occupational Therapy, Fu Jen Catholic University, New Taipei City, Taiwan, Republic of China; Department of General Surgery, Shin Kong Wu Ho-Su Memorial Hospital, Taipei, Taiwan, Republic of China

**Keywords:** recurrent breast cancer, machine learning, classification, AdaBoost, cost-sensitive method

## Abstract

Breast cancer is one of the most common cancers in women all over the world. Due to the improvement of medical treatments, most of the breast cancer patients would be in remission. However, the patients have to face the next challenge, the recurrence of breast cancer which may cause more severe effects, and even death. The prediction of breast cancer recurrence is crucial for reducing mortality. This paper proposes a prediction model for the recurrence of breast cancer based on clinical nominal and numeric features. In this study, our data consist of 1,061 patients from Breast Cancer Registry from Shin Kong Wu Ho-Su Memorial Hospital between 2011 and 2016, in which 37 records are denoted as breast cancer recurrence. Each record has 85 features. Our approach consists of three stages. First, we perform data preprocessing and feature selection techniques to consolidate the dataset. Among all features, six features are identified for further processing in the following stages. Next, we apply resampling techniques to resolve the issue of class imbalance. Finally, we construct two classifiers, AdaBoost and cost-sensitive learning, to predict the risk of recurrence and carry out the performance evaluation in three-fold cross-validation. By applying the AdaBoost method, we achieve accuracy of 0.973 and sensitivity of 0.675. By combining the AdaBoost and cost-sensitive method of our model, we achieve a reasonable accuracy of 0.468 and substantially high sensitivity of 0.947 which guarantee almost no false dismissal. Our model can be used as a supporting tool in the setting and evaluation of the follow-up visit for early intervention and more advanced treatments to lower cancer mortality.

## 1 Introduction

Breast cancer is one of the most common invasive cancers nowadays. According to the World Health Organization (WHO) report in 2018, the breast cancer is the most frequent cancer among women [1]. It impacts 2.1 million women each year and causes the most significant number of deaths among all types of cancers. In 2018, it is reported that an approximate of 627,000 women, 15% of all cancer deaths among women, died from breast cancer [1]. Moreover, according to the American Cancer Society, from 2007 to 2016, invasive female breast cancer incidence rate increased slightly by 0.3% per year. The female breast cancer death rate peaked at 33.2 (per 100,000) in 1989 and declined by 40% to 19.8 in 2017, which was still a high-rate mortality [2]. In Taiwan, breast cancer has the fourth cancer mortality and remains the highest cancer incidence rate in women in 2014.

More and more studies indicate that screening methods, including mammography, ultrasound, and MRI, may reduce breast cancer mortality and also increase the survival rate of breast cancer [3,4].

The mortality of breast cancer can be reduced by 40% for those who take part in screening every 1–2 years [5,6]. Besides, for those diagnosed with breast cancer, patients would be in remission because of earlier detection and improved treatment. According to a survey of breast cancer statistics in 2019 [7], the average 5-year survival rate is approximately 90%, and the average 10-year survival rate is 83%.

Although breast cancer can be in remission by early detection and improved medical techniques, some patients suffer from breast cancer recurrence. Breast cancer recurrence is a fundamental clinical manifestation and it even is the primary cause of breast cancer-related deaths [8]. In recent years, many researchers have tried to find a particular pattern predicting breast cancer recurrence [9]. For instance, by characterizing the presence of breast cancers’ receptors, including ER, PR, HER2, and TNBCs, each subtype will have a higher risk of recurrence than others during particular years or in a specific situation [10,11,12]. Furthermore, axillary lymph node metastases are related to breast cancer recurrence [13]. The chances of breast cancer recurrence can be reduced by intervening in the metastases in an early stage. However, these patterns demand considerable cost and are time-consuming.

As a result, we would like to propose a noninvasive computational model to predict the risk of the recurrence of breast cancer. Like [14,15], we make use of patients’ clinical and treatment information in Breast Cancer Registry to build a prediction model and evaluate various approaches to achieve our goal. Compared with the patterns mentioned in prior, our model can be used in a clinical application after the treatment of original breast cancer in a low-cost and time-saving setting.

In the medical field, Machine Learning (ML) approaches are emerging techniques to resolve medical issues. For instance, Chen et al. develop an early prediction method which makes use of three-year hospital data to effectively predict chronic disease outbreaks. In the study, Chen et al. utilize both structured data and unstructured data [16]. In another study [17], the author proposes a general disease forecasting approach using the symptoms of the patient. The study utilizes K-Nearest Neighbor and convolutional neural network to predict the disease. Moreover, some significant research studies implement ML algorithms to forecast the recurrence of breast cancer. For instance, the study [15] implements three ML algorithms, including artificial neural networks (ANN), decision tree (DT), and Support Vector Machine (SVM) for breast cancer prediction. The study utilizes the Iranian Center breast cancer data for the prediction. The dataset consists of 1,189 records with 22 predictor variables and also a single outcome variable. In the study, the SVM outperforms other techniques and scores the highest accuracy and minimum error rate. In the study [18], the authors apply the NLP and ML algorithms to obtain features of breast cancer and organize the dataset as a comprehensive database. The study collects data from the King Abdullah University Hospital (KAUH) in Jordan. The data consist of 1,475 patient records which hold 142 breast cancer cases. Subsequently, the authors build a model for predicting the recurrence of breast cancer for choosing proper treatment methods and therapy. The research indicates that the bagging classifier outperforms other classifiers and scores an accuracy of 0.923 and a sensitivity of 0.923 [18]. In the study [19], the authors identify the elements significantly associated with recurrent breast cancer and employ the ANN model to detect the recurrence within ten years after breast cancer surgery. A total of 1,140 patients data is involved in this study. The model scores an accuracy of 0.988 and a sensitivity of 0.954. The research [20] utilizes the DT C5.0 to achieve early detection of recurrent breast cancer. A total of 5,471 independent records are secured from official statistics of the Ministry of Health and Medical Education and the Iran Cancer Research Center patients with breast cancer. In the study, the authors employ some features such as the LN (Lymph Node) involvement rate, HER2 (Human Epidermal Growth Factor Receptor 2) value, and Tumor size for prediction. The model achieves an accuracy of 0.819 and a sensitivity of 0.869.

## 2 Materials and methods

### 2.1 Dataset

Our dataset has been taken from the Breast Cancer Registry from Shin Kong Wu Ho-Su Memorial Hospital between 2011 and 2016. This dataset consists of 1,061 patients and 85 clinical features, as shown in Appendix 1. Furthermore, merely 37 records, approximately 3.5%, have a recurrence; the data appear to be extremely imbalanced.

Since some particular values represent unfilled fields or inapplicable values, we perform data cleaning to replace those values as missing values. We then perform data preprocessing on the features of “*smoking behavior,*” “*betel nut chewing behavior,*” and “*drinking behavior*” from a complex nominal data to binary class data in which Class 1 indicates having this behavior and Class 0 denotes oppositely. We also transfer the target feature of “*recurrence*” from date format to a ‘YES’ or ‘NO’ binary class. To be more specific, if there is a date value, we regard it as ‘YES’; otherwise, ‘NO’.

Moreover, another data mining technique has been used – data integration. It involves combining data from several features and provide a unified view of data. We employ the feature, Body Mass Index (BMI), by integrating height and weight. The formula is:

(1) \text{BMI}=\frac{\text{Weight}\hspace{.5em}\text{(kg)}}{\text{Height}\hspace{.5em}{\text{(m}}^{2}\text{)}}]

According to the Breast Cancer Registry, there are seven different therapies (i.e., Surgery, RT, Chemotherapy, Hormone/Steroid Therapy, Immunotherapy, Hematologic Transplant and Endocrine Procedure, and Target Therapy), and each of the therapies could be received in the declaration facility or others. In order to observe the relationship between these therapies and recurrence, we first integrate the corresponding features to define seven features that could indicate whether the patient had received this therapy or not. In reference to Appendix 1, we integrate (23)–(32) and (37) to Surgery, (33)–(52) to RT, (53)–(55) to Chemotherapy, (56)–(58) to Hormone/Steroid Therapy, (59)–(61) to Immunotherapy, (62)–(63) to Hematologic Transplant and Endocrine Procedure, and (64)–(66) to Target Therapy. Note that we remove the Hematologic Transplant and Endocrine Procedure since it is not available in the declaration facility or others. As a result, we have six remaining kinds of therapies in this study, as well as the corresponding user-defined features. Then, we perform data preprocessing: If the result turns out to be “YES,” we would give a value of 1 in the field; otherwise, value of 0 will be given.

Moreover, we transform the 14 date-related features into the 12 duration features. To be more specific, we take the 11 date-related features as the “start date,” including *Date of First Contact*, *Date of Initial Diagnosis*, *Date of First Microscopic Confirmation*, *Date of First Course of Treatment*, *Date of First Surgical Procedure*, *Date of Most Definite Surgical Resection of the Primary Site*, *Date of Chemotherapy Started at This Facility*, *Date of Hormone/Steroid Therapy Started at This Facility*, *Date of Immunotherapy Started at This Facility*, *Date of HT and EP Started at This Facility,* and *Date of Target Therapy Started at This Facility*, and regard *Date of Last Contact* or *Death* as the “end date” to calculate the 11 duration features. Similarly, we calculate the difference between the remaining two features, *Date of RT Started* and *Date of RT Ended*, to define the duration of RT. The mean and standard deviation of duration features are shown in Table 1.

**Table 1:** The statistics of duration features

Features	Total (*n* = 1,061)
Duration of first contact	1.88 ± 1.31
Duration of initial diagnosis	0.94 ± 1.28
Duration of first microscopic confirmation	0.93 ± 1.29
Duration of first course of treatment	1.17 ± 1.36
Duration of first surgical procedure	1.13 ± 1.36
Duration of most definite surgical resection of the primary site	1.13 ± 1.35
Duration of RT (days)	42.61 ± 6.37
Duration of chemotherapy started at this facility	1.16 ± 1.37
Duration of hormone/steroid therapy started at this facility	1.06 ± 1.33
Duration of immunotherapy started at this facility	0.50 ± 0.71
Duration of HT and EP started at this facility	N/A
Duration of target therapy started at this facility	0.98 ± 1.27

*Duration Features are in years, except the Duration of RT.

After applying data transformation techniques, we add *Surgery*, *RT*, *Chemotherapy*, *Hormone*/*Steroid Therapy*, *Immunotherapy*, *Target Therapy*, *BMI*, *Duration of First Contact*, *Duration of Initial Diagnosis*, *Duration of First Microscopic Confirmation*, *Duration of First Course of Treatment*, *Duration of First Surgical Procedure*, *Duration of Most Definite Surgical Resection of the Primary Site*, *Duration of RT (days)*, *Duration of Chemotherapy Started at This Facility*, *Duration of Hormone/Steroid Therapy Started at This Facility*, *Duration of Immunotherapy Started at This Facility*, *Duration of HT and EP Started at This Facility,* and *Duration of Target Therapy Started at This Facility* and remove *Height*, *Weight*, *Date of First Contact*, *Date of Initial Diagnosis*, *Date of First Microscopic Confirmation*, *Date of First Course of Treatment*, *Date of First Surgical Procedure*, *Date of Most Definite Surgical Resection of the Primary Site*, *Date of RT Started*, *Date of RT Ended*, *Date of Chemotherapy Started at This Facility*, *Date of Hormone/Steroid Therapy Started at This Facility*, *Date of Immunotherapy Started at This Facility*, *Date of HT* and *EP Started at This Facility*, *Date of Target Therapy Started at This Facility,* and *Date of Last Contact or Death*. The number of original features will be 85, while the number of features after preprocessing will be 89 and are shown in Appendixes 1 and 2, respectively.

It is noted that features such as *Reasons for No RT* or *Reason for No Surgery of Primary Site* are not descriptive data, they have already been categorized to nominal data. Take *Reason for No RT* as example, there are 8 classes to define this feature, noted as ‘0’, ‘1’, ‘2’, ‘5’, ‘6’, ‘7’, ‘8’, and ‘9’. Each class has its definition. For instance, ‘1’ represents RT is not the priority treatment for the current patient, and ‘5’ denotes current patient expired before having RT.

**Figure 1:**
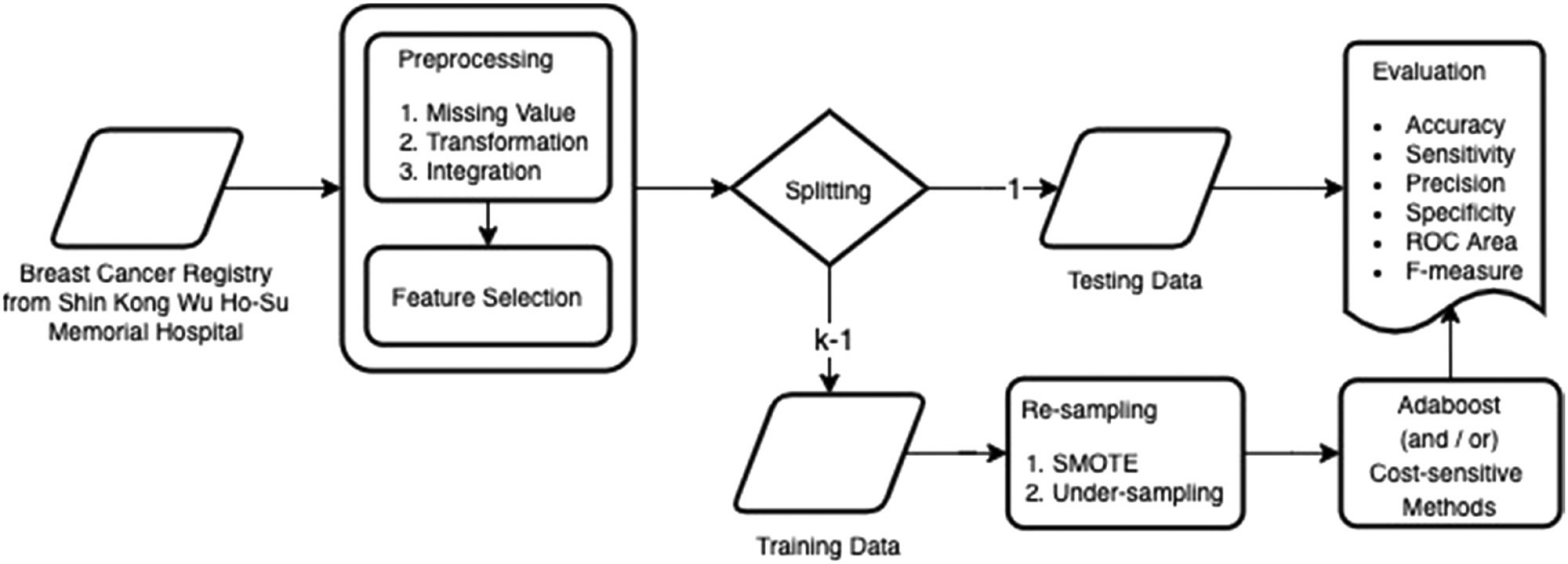
The approach and the process flow of our system architecture.

Furthermore, we introduce the approach and illustrate the process flow of the system architecture in Figure 1. Starting from the left-hand side, we perform data preprocessing, including handle missing values, data transformation, and data integration (detailed in this section), and feature selection (detailed in Section *Feature Selection*) on the dataset. After splitting data into training data and testing data, we apply resampling techniques, SMOTE and Under-sampling, on training data to solve the problem of data imbalance (detailed in Section *Resampling*). We then apply two classification algorithms, AdaBoost and Cost-sensitive learning, to build our model (detailed in Section *Classification Algorithm*). Finally, we employ the *k*-fold cross-validation to evaluate the results with six metrics, including accuracy, sensitivity, precision, specificity, ROC area, and *F*-measure (detailed in Section *Evaluation*).

### 2.2 Feature selection

We apply the feature selection approach including the Correlation-based Feature Selector and the Best First Search to reduce the computation overhead of massive data.

The Correlation-based Feature Selector (CFS) is a filter algorithm that evaluates the worth of feature subsets in which the subsets are highly correlated with the class and having low intercorrelation at the same time. The CFS is based on a correlation-based heuristic evaluation function and the feature that is accepted depending on the entire instance.

The Best First Search (BestFirst) is a searching strategy that searches the space of feature subsets by greedy hill-climbing and a backtracking ability. The BestFirst moves through the entire space by deciding on the present feature subset; once the promising of the path decreases, the feature subset will backtrack to the previous subset and proceed with the task.

When implementing a preprocessing step for ML, this assembly of feature selection, CfsSubsetEval and BestFirst, has been found to perform the best [21].

### 2.3 Resampling

As the recurrence of the breast cancer dataset is imbalanced, we apply resampling techniques on training data to handle the disproportionate ratio of observations in each class and to enhance the class boundaries. In our experiments, we perform Under-sampling and Synthetic Minority Over-sampling Technique (SMOTE) [22] in several different proportions. Under-sampling removes some observations of the majority class, while SMOTE generates new and synthetic data by using the nearest neighbor’s algorithm.

### 2.4 Classification algorithm

Various kinds of ML algorithms solve the classification tasks. According to a prestigious ML competition, KDD Cup, the ensemble method placed first in last 13 years (2005–2018) [23,24,25,26,27,28,29,30,31]. It also dominated in other competitions, the Netflix Competition [32] and Kaggle [33]. The ensemble method improves performance by combining several base learners into one prediction model. This result is also applied in our previous paper [4]. Moreover, the ensemble method has been proved to be robust to handle class imbalance [34,35,36,37,38], which also appeared in this study of Breast Cancer Registry.

Among all ensemble learning algorithms, AdaBoost [39] (Adaptive Boosting), proposed by Freund and Schapire, is one of the most important algorithms. According to a study in [40], AdaBoost has a solid theoretical foundation, which produces extremely accurate prediction with incredible simplicity and has a wide range of successful applications. Furthermore, AdaBoost is robust, which dominates over outliers or noisy data and avoids overfitting problems, so it is also known as the best out-of-the-box classifier [41,42]. The AdaBoost combines the classifiers from the weak learners on various distributions to make itself strong and thus drastically improves the performance. Therefore, we choose AdaBoost as our classifier algorithm to achieve better performance.

The cost-sensitive method [43,44,45] is a type of learning in data mining which aims to get minimal cost class results on an imbalanced dataset. By re-weighting the cost matrix, the classifier will attempt to make decisions on the fewer weight cases and avoid predicting the high-cost cases. In our experiments, we expect the model to make fewer error predictions on the recurrence class, which is the false-negative case. Since the consequences of the misjudgment for facing the recurrence would be too expensive, a higher penalty will be given to the weight of the false-negative case in order to achieve approximately 100% sensitivity.

### 2.5 Evaluation

In the experiments, we employ *k*-fold cross-validation to evaluate the performance of the model. We first randomly divide the dataset into *k* equal sized partitions. For each unique partition, we take it as the validation dataset for evaluating the model, and the remaining (*k* −1) subsamples are considered the training dataset. Afterwards, average the results from the *k* times process of cross-validation. In our work, we set *k* as 3. The first fold includes 342 no-recurrent and 12 recurrent records, the second fold contains 341 no-recurrent and 13 recurrent records, and the third fold consists of 341 no-recurrent and 12 recurrent records. Moreover, accuracy, sensitivity, precision, specificity, ROC area, and *F*-measure will be reported to evaluate model performance and defined as follows. We use the confusion matrix, shown in Table 2, to describe the evaluation metrics for better understanding.

- Accuracy measures the ratio of correct predictions over all evaluated cases.
  (2) \text{Accuracy}=\frac{\text{TP}+\text{TN}}{\text{TP}+\text{FN}+\text{FP}+\text{TN}}]
- Sensitivity measures the fraction of positive actual cases that are correctly predicted.
  (3) \text{Sensitivity}=\frac{\text{TP}}{\text{TP}+\text{FN}}\text{ }]
- Precision measures the proportion of positive predictions that are positive actual cases.
  (4) \text{Precision}=\frac{\text{TP}}{\text{TP}+\text{FP}}]
- Specificity measures the fraction of negative actual cases that are correctly predicted.

(5) \text{Specificity}=\frac{\text{TN}}{\text{FP}+\text{TN}}]

- ROC area stands for “Receiver Operating Characteristic Area,” also known as “Area Under the ROC Curve” (AUC). It measures the performance as a relative trade-off between Sensitivity and Specificity.
- *F*-measure is the harmonic mean of Sensitivity and Precision. The higher the *F*-measure, the better the predictive power of the model.

(6) F\text{-measure}\hspace{.25em}=\frac{2}{\frac{1}{\text{Sensitivity}}+\frac{1}{\text{Precision }}}=2\times \frac{\text{Sensitivity}\times \text{Precision }}{\text{Sensitivity}+\text{Precision}}]

**Table 2:** The confusion matrix

	Positive prediction	Negative prediction
Positive actual class	True positive (TP)	False negative (FN)
Negative actual class	False positive (FP)	True negative (TN)

## 3 Results

Our approach consists of three stages. We first perform data preprocessing and feature selection which have been detailed in Section *Dataset* and *Feature Selection*, respectively. The statistics of selected features of our dataset are shown in Table 3. Moreover, the six-selected features are described as follows:

- Regional Lymph Nodes Positive records the total number of regional lymph nodes tested positive by the pathologist. It can be used to evaluate the quality of a pathology report, the extent of surgery, and the measurement of treatment quality.
- Duration of First Contact is a feature of duration between *Date of Last Contact* or *Death* and *Date of First Contact*. It is important information for clinical examinations to follow up on the recurrence of breast cancer.
- Tumor Size describes the maximum size of the primary tumor in millimeters. (rounded to the nearest millimeter)
- Cancer Status records the existence of cancer before the *Date of Last Contact* or *Death*. There are two classes in this feature, including ‘no evidence of the existence of this primary cancer’ and ‘the presence of this primary cancer.’
- Response to Neoadjuvant Therapy describes the response of breast cancer cases after receiving Neoadjuvant Therapy. There are six classes in this feature, including ‘complete response (CR),’ ‘moderate response (PR),’ ‘poor response (PD),’ ‘w/o Neoadjuvant Therapy,’ ‘w/o Neoadjuvant Therapy,’ and ‘N/A (missing value).’
- Clinical N refers to whether there is regional lymph node metastasis and the scope of metastasis. It is used to carry out prognosis estimation, treatment planning, evaluation of new therapies, result analysis, follow-up planning, and early detection results evaluation. There are 11 classes in this feature, including ‘NX,’ ‘N0,’ ‘N1,’ ‘N2,’ ‘N2a,’ ‘N3,’ ‘N3a,’ ‘N3b,’ ‘N3c,’ ‘no suitable definition,’ and ‘N/A (missing value).’

**Table 3:** The statistics of selected features by the feature selection algorithm

Variable	Total	Nonrecurrent	Recurrent
	*n* = 1,061	*n* = 1,024	*n* = 37
Regional lymph nodes positive	1.24 ± 3.36	1.12 ± 3.21	3.53 ± 5.07
Duration of first contact (year)	1.88 ± 1.31	1.83 ± 1.29	3.08 ± 1.32
Tumor size (mm)	26.23 ± 21.8	25.34 ± 18.94	49.16 ± 54.89
**Cancer status**	(100%)	(100%)	(100%)
No evidence of the existence of this primary cancer	753 (71.0%)	743 (72.6%)	10 (27.0%)
The presence of this primary cancer	308 (29.0%)	281 (27.4%)	27 (73.0%)
**Response to Neoadjuvant therapy**	(100%)	(100%)	(100%)
Complete response	13 (1.2%)	11 (1.1%)	2 (5.4%)
Moderate response	2 (0.2%)	2 (0.2%)	0 (0.0%)
Poor response	18 (1.7%)	15 (1.5%)	3 (8.1%)
w/o Neoadjuvant therapy	951 (89.6%)	928 (90.6%)	23 (62.2%)
w/o response	44 (4.1%)	38 (3.7%)	6 (16.2%)
N/A (missing value)	33 (3.1%)	30 (2.9%)	3 (8.1%)
**Clinical N**	(100%)	(100%)	(100%)
NX	26 (2.5%)	26 (2.5%)	0 (0.0%)
N0	747 (70.4%)	732 (71.5%)	15 (40.5%)
N1	193 (18.2%)	184 (18.0%)	9 (24.3%)
N2	40 (3.8%)	32 (3.1%)	8 (21.6%)
N2a	3 (0.3%)	2 (0.2%)	1 (2.7%)
N3	8 (0.8%)	7 (0.7%)	1 (2.7%)
N3a	1 (0.1%)	1 (0.1%)	0 (0.0%)
N3b	1 (0.1%)	0 (0.0%)	1 (2.7%)
N3c	7 (0.7%)	7 (0.7%)	0 (0.0%)
No suitable definition	3 (0.3%)	2 (0.2%)	1 (2.7%)
N/A (missing value)	32 (3.0%)	31 (3.0%)	1 (2.7%)

*Data are presented as number (%) or mean ± std dev.

There are three stages in our approach. The first stage is data preprocessing and feature extraction. Among the eighty-eight features, the six features (including *Regional Lymph Nodes Positive, Duration of First Contact, Tumor Size, Cancer Status, Response to Neoadjuvant Therapy, Clinical* N) are selected. We are wondering whether the model of using only six-selected features downgrades the performance of prediction model. As a result, we provide the Table 4 to support our methodology.

**Table 4:** Performance of all features vs six-selected features by using AdaBoost

# Of features	Accuracy	Sensitivity	Precision	Specificity	ROC Area	*F-*measure
All features	0.972	0.352	0.700	0.994	0.760	0.610
Six-selected features	0.969	0.137	0.917	0.999	0.912	0.238

In reference to Table 4, we summarize the performance of prediction model by using all features and six-selected features. Considering the accuracy, both models are almost the same. Moreover, the model of six features achieves higher precision and ROC area, but lower sensitivity.

Take the results as input for the next stage. In the second stage, we implement different ratios of resampling techniques, including under-sampling and SMOTE, and apply AdaBoost to construct the model.

The second stage results are shown in Table 5. As the ratio of recurrence to no-recurrence is three to one, the *F*-measure is 0.657 which is the highest among all experiments, and the accuracy and sensitivity is 0.973 and 0.675, respectively.

**Table 5:** Applying AdaBoost with resampling techniques

Method	no-R/R	Accuracy	Sensitivity	Precision	Specificity	ROC Area	*F*-measure
w/o resampling techniques	28:1	0.969	0.137	**0.917**	**0.999**	**0.912**	0.238
SMOTE (2)	14:1	0.968	0.222	0.421	0.995	0.911	0.291
SMOTE (4)	7:1	0.977	0.541	0.759	0.993	0.888	0.632
SMOTE (8)	3.5:1	0.974	0.622	0.686	0.986	0.889	0.652
SMOTE (16)	1.7:1	0.968	**0.675**	0.601	0.978	0.900	0.636
SMOTE (8) w/U-S (15)	3:1	**0.973**	**0.675**	0.640	0.983	0.890	**0.657**
SMOTE (8) w/U-S (30)	2.5:1	0.970	**0.675**	0.617	0.981	0.894	0.644

^1^ no-R/R: the ratio of no-recurrent to recurrent.

^2^ SMOTE (*m*): using SMOTE on the minority group by a factor of *m* times.

^3^ U-S (*n*): applying under-sampling to reduce the majority group by *n* percent.

The bold values are the largest values with respect to the corresponding column.

In the third stage, we combine AdaBoost and cost-sensitive methods to build a model with high sensitivity and acceptable accuracy. The performance of the third stage is reported in Table 6. Our model achieves accuracy of 0.468 and sensitivity of 0.947.

**Table 6:** Performance of combing AdaBoost and cost-sensitive methods

Penalty	Accuracy	Sensitivity	Precision	Specificity	ROC Area	*F*-measure
1	0.973	0.675	0.640	0.983	0.890	0.657
10	0.811	0.754	0.143	0.813	0.897	0.241
20	0.710	0.810	0.091	0.707	0.886	0.163
30	0.715	0.835	0.094	0.711	0.888	0.169
40	0.692	0.891	0.093	0.685	0.875	0.168
50	0.665	0.891	0.086	0.656	0.882	0.157
60	0.665	0.891	0.086	0.656	0.881	0.157
70	0.638	0.891	0.080	0.629	0.900	0.147
80	0.599	0.891	0.073	0.589	0.900	0.135
90	0.577	0.891	0.070	0.565	0.900	0.130
100	0.506	0.919	0.063	0.491	0.900	0.118
110	0.543	0.919	0.067	0.529	0.907	0.125
120	0.543	0.919	0.067	0.529	0.907	0.125
130	0.468	**0.947**	0.061	0.450	0.907	0.114
140	0.505	0.919	0.062	0.490	0.894	0.117
150	0.502	0.919	0.062	0.487	0.894	0.116

In the medical application of imbalanced data, it is challenging to build a prediction model of having both high sensitivity and precision. There is a trade-off between sensitivity and precision.

Therefore, in this study, we provide two alternatives of achieving high sensitivity and high precision, respectively. First, we build a prediction model of having high precision by using only the six features, as shown in Table 4. Then, we build a prediction model with resampling techniques, as shown in Table 5.

However, with respect to cancer recurrence prediction, the prediction model would be expected to have high sensitivity but reasonable precision. The cost of misclassification of false negative might not be affordable. As a result, we build another prediction model of having high sensitivity by using cost-sensitive learning methods, to guarantee almost no false dismissal of recurrence prediction, as shown in Table 6.

When dealing with the class imbalance problem in the medical application, we may make use of the cost-sensitive learning algorithm by setting a cost matrix which encodes the *penalty* of misclassification. A cost-sensitive classification technique takes the unequal cost matrix into consideration during model construction and generate a model of the lowest cost. In this study, the penalty is the cost of committing false negative error.

The setting of penalty in the cost-sensitive method is reasonable when applying prediction algorithms in the medical applications, since it would not be affordable for the false negative case. The ‘recurrence cases’ are rare cases, but cannot be missed in the context of prediction. In the medical prediction, the false negative errors are most costly. In this study, we make use of cost-sensitive methods to reduce the errors by extending their decision boundary toward the negative class, in order to achieve a high sensitivity.

In reference to Table 6, as setting the penalty of 130, the sensitivity is 0.947 and the ROC area is 0.907. That is, our proposed method would guarantee almost “no false dismissal,” although it may raise some false alarms.

## 4 Discussion

Some discussions based on the experiment results are given below. First of all, our study employs data preprocessing and integration to obtain information for clinical examination. Moreover, we apply feature extraction algorithms to determine most essential features among all features in our dataset of Breast Cancer Registry. As a result, the six features shown in Table 3 are chosen in which the *Duration of the First Contact* is also selected by the feature selection algorithm. The selected features conform to Dr. Chung-Ho Hsieh’s clinical experience in the recurrence of breast cancer. In addition, six-selected features achieve almost the same performance of using all features in terms of accuracy.

The *Duration of First Contact* could be approximately interpreted as the “Disease-Free Interval” which is one of risk factors of cancer recurrence [46]. In addition, according to [10], the risk of breast cancer recurrence will reach a peak in the first two years and then decrease gradually. Meanwhile, the average *Duration of First Contact* with respect to the ‘Recurrent’ patients is 3.08 years in our dataset. The slight difference between the two reports will be further studied in our future work.

In Section 1, we quote “For instance, by characterizing the presence of breast cancers’ receptors, including ER, PR, HER2 and TNBCs, each subtype will have a higher risk of recurrence than others during particular years or in a specific situation [10,11,12].”

In our study, we have investigated the performance of prediction model by using all features first. The features of ER, PR, HER2, and tumor size are also included in our dataset of breast cancer registry. Then, by applying the feature selection procedure, the six features are chosen to achieve better performance (in terms of ROC area and precision) without sacrificing accuracy.

In addition, we perform experiments by using ER, PR, and HER2. Note that we do not have TNBCs in our dataset of breast cancer registry. According to our experiment results, the accuracy of using ER, PR, and HER2 is not as good as those of using the all features or six-selected features. In more detail, when applying the model of using ER, PR, and HER2, all instances are classified as negative cases. That is, the model has no predictive power.

The resampling techniques play a crucial role while building the model for imbalanced data. The study utilizes various approaches to tackle the variance of the dataset. Initially, we have implemented SMOTE to reduce the variance of the dataset. Ensemble methods are also an alternative approach to handle this imbalanced dataset. Accordingly, to construct a strong model, we have employed the AdaBoost ensemble method.

Applying the cost-sensitive method shows the trade-off between accuracy and sensitivity. In the beginning, as we set equal cost, the accuracy is 0.973 and the sensitivity is 0.675. If we slightly increase the penalty of the cost-sensitive algorithm, the accuracy will be down to 0.811 and the sensitivity will be up to 0.754. When the penalty is set to 130, our model has the sensitivity of 0.947.

In the third stage, we meet our goal of building a prediction model with high sensitivity and reasonable accuracy in order to assist the early diagnosis, treatment choice, and determination of follow-up visit frequency. Recently, some approaches have been proposed for recurrence prediction of breast cancer, which are described in the Section 1. In reference to Table 7, we summarize the performance of breast cancer recurrence prediction methods. At first glance, it seems that our approach does not outperform the ANN [19]. However, we would like to point out that our dataset is highly imbalanced. The percentage of recurrence in our dataset is 3.5%; the baseline of our dataset is considerably high. The “baseline” is calculated by dividing the number of data in the category with the largest number by the total number of the dataset. From the perspective of performance over baseline, our approach performs well with respect to the highly imbalanced dataset.

**Table 7:** Performance comparison of breast cancer recurrence prediction model

Method	Accuracy	Sensitivity	Selected features	Dataset size (total/# of recurrence)
BCRSVM [14]	0.846	0.890	Histological grade, local invasion of tumor, no of tumors, tumor size, LVI, ER, no of metastatic lymph nodes	679/195 (29%)
SVM [15]	0.957	0.971	Age at diagnosis, age at menarche, age at menopause, tumor Size, LN involvement, grade, nexion (lymph node dissection), HER2	547/117 (21%)
Bagging [18]	0.923	0.923	Tumor grade, molecular subtype, cancer focality, LVI, menopause, DCIS type, age, and dimension of primary tumor	1,475/142 (10%)
OneR [18]	0.901	0.901		
ANN [19]	0.988	0.954	Surgeon volume, hospital volume, tumor stage	1,140/225 (20%)
SVM [19]	0.897	0.704		
KPCA-SVM [20]	0.785	0.833	LN involvement rate, HER2 value, tumor size, tumor margin.	5,471/2,517 (46%)
C5.0 [20]	0.819	0.869		
AdaBoost	0.973	0.675	Regional lymph nodes positive, duration of first contact, tumor size (mm), cancer status, response to Neoadjuvant therapy, clinical N	1,061/37 (3.5%)
AdaBoost + cost-sensitive method	0.468	0.947		

## 5 Conclusion

This paper proposes a ML approach to build a noninvasive computational model for predicting the risk of breast cancer recurrence using imbalanced data. As the result, our models could be able to serve in a clinical application of early diagnosis, to predict the risk of the recurrence after the treatment of original breast cancer. Early prediction can help with early diagnosis and prevention of the cancer recurrence. Based on our model, physicians can take the prediction results as reference in deciding treatment methods that provide extra support for better decision making.

We use patients’ clinical data and solve the problem of data imbalance by employing resampling techniques, cost-sensitive learning, and ensemble methods. We construct two prediction models. The first model performs a high accuracy and reasonable sensitivity, while the second model performs oppositely. With our approach, the first model is able to achieve accuracy of 0.973 and sensitivity of 0.675 and the second model guarantees almost “no false dismissals,” which means the sensitivity is approximately 100%. The accuracy and sensitivity will be 0.468 and 0.947, respectively.

## Appendix 1 The 85 original features of patient records of the Breast Cancer Registry

**Table:**

Feature categories	Features
Case confirmation	(1) Sex
	(2) Date of birth
Cancer confirmation	(3) Age at diagnosis
	(4) Sequence number
	(5) Date of first contact
	(6) Date of initial diagnosis
	(7) Primary site
	(8) Laterality
	(9) Histology
	(10) Behavior code
	(11) Grade/differentiation
	(12) Diagnostic confirmation
	(13) Date of first microscopic confirmation
	(14) Tumor size
	(15) Regional lymph nodes examined
	(16) Regional lymph nodes positive
Stage of disease at initial diagnosis	(17) Clinical T
	(18) Clinical N
	(19) Clinical M
	(20) Clinical stage group
	(21) Clinical stage (prefix/suffix) descriptor
Treatment	(22) Date of first course of treatment
	(23) Date of first surgical procedure
	(24) Date of most definite surgical resection of the primary site
	(25) Surgical procedure of primary site at other facility
	(26) Surgical procedure of primary site at this facility
	(27) Surgical margins of the primary site
	(28) Scope of regional lymph node surgery at other facility
	(29) Scope of regional lymph node surgery at this facility
	(30) Surgical procedure/other site at other facility
	(31) Surgical procedure/other site at this facility
	(32) Reason for No Surgery of Primary Site
	(33) RT target summary
	(34) RT technique
	(35) Date of RT started
	(36) Date of RT ended
	(37) Sequence of radiotherapy and surgery
	(38) Sequence of locoregional therapy and systemic therapy
	(39) Institute of RT
	(40) Reasons for No RT
	(41) EBRT instruments
	(42) Target of CTV_H
	(43) Dose to CTV_H (cGy)
	(44) Number of Fractions to CTV_H
	(45) Target of CTV_L
	(46) Dose to CTV_L (cGy)
	(47) Number of fractions to CTV_L
	(48) Other RT technique
	(49) Other RT instruments
	(50) Target of other RT
	(51) Dose to target of other RT
	(52) Number of fractions to other RT
	(53) Chemotherapy at other facility
	(54) Chemotherapy at this facility
	(55) Date of chemotherapy started at this facility
	(56) Hormone/steroid therapy at other facility
	(57) Hormone/steroid therapy at this facility
	(58) Date of hormone/steroid therapy started at this facility
	(59) Immunotherapy at other facility
	(60) Immunotherapy at this facility
	(61) Date of immunotherapy started at this facility
	(62) Hematologic transplant and endocrine procedure
	(63) Date of HT and EP started at this facility
	(64) Target therapy at other facility
	(65) Target therapy at this facility
	(66) Date of target therapy started at this facility
	(67) Palliative care at this facility
Treatment result	(68) Vital status
	(69) Cancer status
	(70) Recurrence (*Target in our study*)
	(71) Date of last contact or death
Breast cancer site-specific factors	(72) Estrogen receptor assay
	(73) Progesterone receptor assay
	(74) Response to Neoadjuvant therapy
	(75) No. of sentinel lymph nodes examined
	(76) No. of sentinel lymph nodes positive
	(77) Nottingham or Bloom-Richardson (BR) score/grade
	(78) HER2 (human epidermal growth factor receptor 2) IHC test lab value
	(79) Paget disease
	(80) Lymph vessels or vascular invasion (LVI)
Other factors	(81) Height
	(82) Weight
	(83) Smoking behavior
	(84) Betel net chewing behavior
	(85) Drinking behavior

## Appendix 2 The 88 features after data preprocessing

**Table:**

Feature categories	Features
Case confirmation	(1) Sex
	(2) Age (Date of birth)
Cancer confirmation	(3) Age at diagnosis
	(4) Sequence number
	(5) Duration of first contact
	(6) Duration of initial diagnosis
	(7) Primary site
	(8) Laterality
	(9) Histology
	(10) Behavior code
	(11) Grade/differentiation
	(12) Diagnostic confirmation
	(13) Duration of first microscopic confirmation
	(14) Tumor size
	(15) Regional lymph nodes examined
	(16) Regional lymph nodes positive
Stage of disease at initial diagnosis	(17) Clinical T
	(18) Clinical N
	(19) Clinical M
	(20) Clinical stage group
	(21) Clinical stage (prefix/suffix) descriptor
Treatment	(22) Duration of first course of treatment
	(23) Duration of first surgical procedure
	(24) Duration of most definite surgical resection of the primary site
	(25) Surgery
	(26) Surgical procedure of primary site at other facility
	(27) Surgical procedure of primary site at this facility
	(28) Surgical margins of the primary site
	(29) Scope of regional lymph node surgery at other facility
	(30) Scope of regional lymph node surgery at this facility
	(31) Surgical procedure/other site at other facility
	(32) Surgical procedure/other site at this facility
	(33) Reason for no surgery of primary site
	(34) RT target summary
	(35) RT technique
	(36) Duration of RT (days)
	(37) RT
	(38) Sequence of radiotherapy and surgery
	(39) Sequence of locoregional therapy and systemic therapy
	(40) Institute of RT
	(41) Reasons for no RT
	(42) EBRT instruments
	(43) Target of CTV H
	(44) Dose to CTV H (cGy)
	(45) Number of fractions to CTV H
	(46) Target of CTV L
	(47) Dose to CTV L (cGy)
	(48) Number of fractions to CTV L
	(49) Other RT technique
	(50) Other RT instruments
	(51) Target of other RT
	(52) Dose to target of other RT
	(53) Number of fractions to other RT
	(54) Chemotherapy at other facility
	(55) Chemotherapy at this facility
	(56) Duration of chemotherapy started at this facility
	(57) Chemotherapy
	(58) Hormone/steroid therapy at other facility
	(59) Hormone/steroid therapy at this facility
	(60) Duration of hormone/steroid therapy started at this facility
	(61) Hormone/steroid therapy
	(62) Immunotherapy at other facility
	(63) Immunotherapy at this facility
	(64) Duration of immunotherapy started at this facility
	(65) Immunotherapy
	(66) Hematologic transplant and endocrine procedure
	(67) Duration of HT and EP started at this facility
	(68) Target therapy at other facility
	(69) Target therapy at this facility
	(70) Duration of target therapy started at this facility
	(71) Target therapy
	(72) Palliative care at this facility
Treatment result	(73) Vital status
	(74) Cancer status
	(75) Recurrence (*Target in our study*)
Breast cancer site-specific factors	(76) Estrogen receptor assay
	(77) Progesterone receptor assay
	(78) Response to Neoadjuvant therapy
	(79) No. of sentinel lymph nodes examined
	(80) No. of sentinel lymph nodes positive
	(81) Nottingham or Bloom-Richardson(BR) score/grade
	(82) HER2 (human epidermal growth factor receptor 2) IHC test lab value
	(83) Paget disease
	(84) Lymph Vessels or Vascular Invasion (LVI)
Other factors	(85) BMI
	(86) Smoking behavior
	(87) Betel net chewing behavior
	(88) Drinking behavior
